# Radiomics for prediction of radiation-induced lung injury and oncologic outcome after robotic stereotactic body radiotherapy of lung cancer: results from two independent institutions

**DOI:** 10.1186/s13014-021-01805-6

**Published:** 2021-04-16

**Authors:** Khaled Bousabarah, Oliver Blanck, Susanne Temming, Maria-Lisa Wilhelm, Mauritius Hoevels, Wolfgang W. Baus, Daniel Ruess, Veerle Visser-Vandewalle, Maximilian I. Ruge, Harald Treuer, Martin Kocher

**Affiliations:** 1 Department of Stereotactic and Functional Neurosurgery, University Hospital of Cologne, Kerpener Str. 62, 50937 Cologne, Germany; 2 Institute of Diagnostic and Interventional Radiology, University Hospital of Cologne, Cologne, Germany; 3 Department of Radiation Oncology, University Medical Center Schleswig-Holstein, Kiel, Germany; 4 Saphir Radiosurgery Center Northern Germany, Guestrow, Germany; 5 Department of Radiation Oncology, University Hospital of Cologne, Cologne, Germany; 6 Department of Radiation Oncology, University Medicine Rostock, Rostock, Germany

## Abstract

**Objectives:**

To generate and validate state-of-the-art radiomics models for prediction of radiation-induced lung injury and oncologic outcome in non-small cell lung cancer (NSCLC) patients treated with robotic stereotactic body radiation therapy (SBRT).

**Methods:**

Radiomics models were generated from the planning CT images of 110 patients with primary, inoperable stage I/IIa NSCLC who were treated with robotic SBRT using a risk-adapted fractionation scheme at the University Hospital Cologne (training cohort). In total, 199 uncorrelated radiomic features fulfilling the standards of the Image Biomarker Standardization Initiative (IBSI) were extracted from the outlined gross tumor volume (GTV). Regularized models (Coxnet and Gradient Boost) for the development of local lung fibrosis (LF), local tumor control (LC), disease-free survival (DFS) and overall survival (OS) were built from either clinical/ dosimetric variables, radiomics features or a combination thereof and validated in a comparable cohort of 71 patients treated by robotic SBRT at the Radiosurgery Center in Northern Germany (test cohort).

**Results:**

Oncologic outcome did not differ significantly between the two cohorts (OS at 36 months 56% vs. 43%, *p* = 0.065; median DFS 25 months vs. 23 months, *p* = 0.43; LC at 36 months 90% vs. 93%, *p* = 0.197). Local lung fibrosis developed in 33% vs. 35% of the patients (*p* = 0.75), all events were observed within 36 months. In the training cohort, radiomics models were able to predict OS, DFS and LC (concordance index 0.77–0.99, *p* < 0.005), but failed to generalize to the test cohort. In opposite, models for the development of lung fibrosis could be generated from both clinical/dosimetric factors and radiomic features or combinations thereof, which were both predictive in the training set (concordance index 0.71– 0.79, *p* < 0.005) and in the test set (concordance index 0.59–0.66, *p* < 0.05). The best performing model included 4 clinical/dosimetric variables (GTV-D_mean_, PTV-D_95%_, Lung-D_1ml_, age) and 7 radiomic features (concordance index 0.66, *p* < 0.03).

**Conclusion:**

Despite the obvious difficulties in generalizing predictive models for oncologic outcome and toxicity, this analysis shows that carefully designed radiomics models for prediction of local lung fibrosis after SBRT of early stage lung cancer perform well across different institutions.

## Introduction

Stereotactic body radiation therapy (SBRT) is an effective therapy for early-stage, node-negative, medically inoperable non-small cell lung cancer (NSCLC). Dose-fractionation schemes usually depend on tumor size and location and have been largely standardized by current guidelines [1–4]. However, after irradiation, about 10–15% of the tumors will recur locally, up to 50% of the patients will experience systemic disease progression despite PET-based staging before SBRT [5], and 25–30% of the patients will develop radiation-induced lung injury (RILI) on follow-up chest imaging. Apart from an established dose–response relationship for local control [6], dosimetric and clinical factors have only shown limited capability in predicting these events [7–14].

Radiomics aims at extraction of biomarkers from high-dimensional analysis of digital images and has been extensively studied in lung cancer by using computed tomography (CT) or Fluor-Deoxyglucose Positron Emission Tomography (FDG-PET) of the chest [15–20]. Several studies have applied radiomic analysis in SBRT of NSCLC [21–34], but so far, the clinical impact of the developed algorithms has been low due to low reproducibility of the results [35], lack of standardization of the extracted radiomic features and lack of external validation on data from other institutions.

The availability of open-source software solutions allows the extraction of standardized radiomic features and generation of complex, non-linear models which are able to account for complex interactions between features and have the potential to achieve high performance. The primary objective of the present study was to build a model for the development of radiation-induced lung injury by use of state-of-the-art feature extraction and machine-learning algorithms in order to determine the extra value of imaging tumor biomarkers when used in addition to dosimetric and clinical factors in a cohort of patients with NSCLC treated by robotic SBRT. Secondary objectives of the study were the development of models for local control, disease free survival and overall survival. The models were trained on data from one institution and tested on a cohort from a separate institution that treated patients based on similar inclusion criteria and fractionation schemes. This work extends an earlier single institution report [36].

## Patients and methods

### Patients, treatment and follow-up

Two cohorts of patients with stage I/IIa NSCLC (according to staging classification of the Union for International Cancer Control [UICC], 8th edition) who underwent definitive robotic SBRT were retrospectively analyzed. The first cohort comprised 110 patients treated at the University Hospital of Cologne, Germany and was used for identification of clinical, dosimetric and image-derived parameters to predict local control (LC), overall survival (OS), disease free survival (DFS) and occurrence of local lung fibrosis (LF) as a manifestation of radiation-induced lung injury after SBRT. This cohort had already been analyzed in a previous study using a simpler radiomics approach that only included first-level features and a small set of 5 texture features from the Gray-Level Co-occurrence Matrix (GLCM) without wavelet-filtering [36] and served as the training data set. A second cohort of 71 patients was treated at the Radiosurgery Center Northern Germany, Guestrow, and was used as test set (in machine learning terminology) for the predictive power of the models developed in the training set.

In both cohorts, patients suffering from a peripheral T1/2 (UICC 8) NSCLC without lymph node metastases who were either medically inoperable or refused resection were treated solely by means of the Cyberknife^R^ system (Accuray, Sunnyvale, USA) without concomitant therapy using a risk-adapted fractionation scheme (peripheral T1 tumors 3 × 13–18 Gy, T1 tumors with broad contact to the chest wall and T2 tumors 5 × 10–11 Gy, near-central or true central tumors 8 × 6–7.5 Gy). The dose was calculated using a Monte Carlo dose calculation algorithm (Multiplan 4.5, Accuray, Sunnyvale, USA) and the prescribed dose was referred to the 65–70% isodose in most cases. The GTV was manually outlined for clinical use on the planning CT, and the PTV was generated by adding a margin of 3-4 mm (Table 1). A set of volumetric and dosimetric parameters was extracted from the planning system including GTV (gross tumor volume), PTV (planning target volume), GTV-D_max_ (maximal dose in GTV), GTV-D_mean_ (mean dose in GTV), GTV-D_95%_ (dose achieved in 95% of the GTV), PTV-D_95%_ (dose achieved in 95% of the PTV) and lung doses Lung-D_1ml_, Lung-D_10ml_, Lung-D_50ml_, Lung-D_100ml_ [13, 37–40], see Table 1. According to the different dose/ fractionation schemes, the doses covered a wide range in both datasets. The GTV-D_max_ which is reported as a point dose from the Cyberknife system might have been biased upwards due to noise induced by the Monte Carlo algorithm, but showed a close correlation with the GTV-D_mean_ in both sets (Pearson correlation coefficient 0.98 and 0.89), indicating that this bias was small. The cohorts also contained 12(8) patients with local stage T1/2 tumors who had been successfully treated for oligo-metastatic disease, and who were free from tumor activity besides the primary tumor. Patient characteristics and treatment parameters are shown in Table 1. All patients had the (3D) planning CT performed under breath-hold conditions which was used for both treatment planning and radiomics image analysis (Table 2).

**Table 1:** Patient and treatment characteristics

	Training set		Test set	
	(*n* = 110)		(*n* = 71)	
Age (median/range)	73y (50–94 year)		75y (48–88 year)	
Gender (male/female)	58/52 (53%/47%)		47/24 (66%/34%)	
Tumor diameter (median/range)	2.2 cm (0.8–6.6 cm)*		2.6 cm (1.1–6.0 cm)^#^	
Tumor stage (UICC8), T1/T2	89/21 (81%/19%)		45/26 (63%/37%)	
Pathological confirmation (Yes/No)	91/19 (83%/17%)		55/16 (77%/23%)	
Mediastinal staging				
CT only	18 (16%)		5 (7%)	
CT + PET	52 (47%)		33 (47%)	
CT + EBUS	18 (16%)		16 (23%)	
CT + EBUS + PET	18 (16%)		17 (24%)	
CT + mediastinoscopy	3 (3%)		–	
CT + PET + mediastinoscopy	1 (1%)		–	
Histology				
Adenocarcinoma	37 (34%)		23 (32%)	
Squamous cell	42 (38%)		28 (39%)	
Other	12 (11%)		4 ( 6%)	
Unknown	19 (17%)		16 (23%)	
*Fractionation scheme*				
Number of fractions	Dose per fraction	n Pat	Dose per fraction	n Pat
1	25 Gy	5 (5%)	26–27 Gy	2 (3%)
3	17 Gy	45 (41%)	13–18 Gy	65 (90%)^§^
5	11 Gy	43 (39%)	10–11 Gy	3 (6%)
8	7.5 Gy	17 (16%)	6.0 Gy	1 (1%)
Doses to GTV, PTV and lung (median/ range)				
GTV D_max_	84.6 (28.2–95.2) Gy		70.9 (41.5–84.6) Gy	
GTV D_mean_	71.6 (26.2–84.0) Gy		62.7 (37.9–72.5) Gy	
GTV D_95%_	61.9 (21.8–75.9) Gy		53.8 (33.0–64.6) Gy	
PTV D_95%_	54.0 (19.0–67.1) Gy		45.3 (25.2–55.2) Gy	
Lung D_1ml_	65.6 (23.6–81.0) Gy		55.5 (37.7–71.6) Gy	
Lung D_10ml_	52.1 (15.8–78.9) Gy		47.5 (25.8–66.9) Gy	
Lung D_50ml_	31.3 (6.9–77.7) Gy		31.6 (11.2–51.9) Gy	
Lung D_100ml_	20.5 (4.5–77.0) Gy		20.5 (6.6–43.0) Gy	
GTV-PTV margin	3–4 mm		3–5 mm	
Tracking Mode (Fiducials/XSightLung)	15/95 (14%/86%)		6/65 (9%/91%)	

*1 pt. > 5 cm

^#^3 pts. > 5 cm

^§^1 pt. 4 × 10 Gy

**Table 2:** Imaging parameters

	Training set	Test set
CT scanner	Aquilion LB-CT, Toshiba	Brilliance 16, Philips
Slice thickness	1.0 mm	1.5 mm
Transversal resolution	0.93–1.37 mm	0.93–0.97 mm
Voltage	120KV	120KV
Current–time product	400mAs	400-450mAs
Image matrix	512 × 512	512 × 512
Reconstruction kernel	FC17	B
Contrast agent	None (84%), Accupaque^R^ 300 (16%)*	None (100%)

*No significant impact on GTV radiodensity

**Fig. 1:**
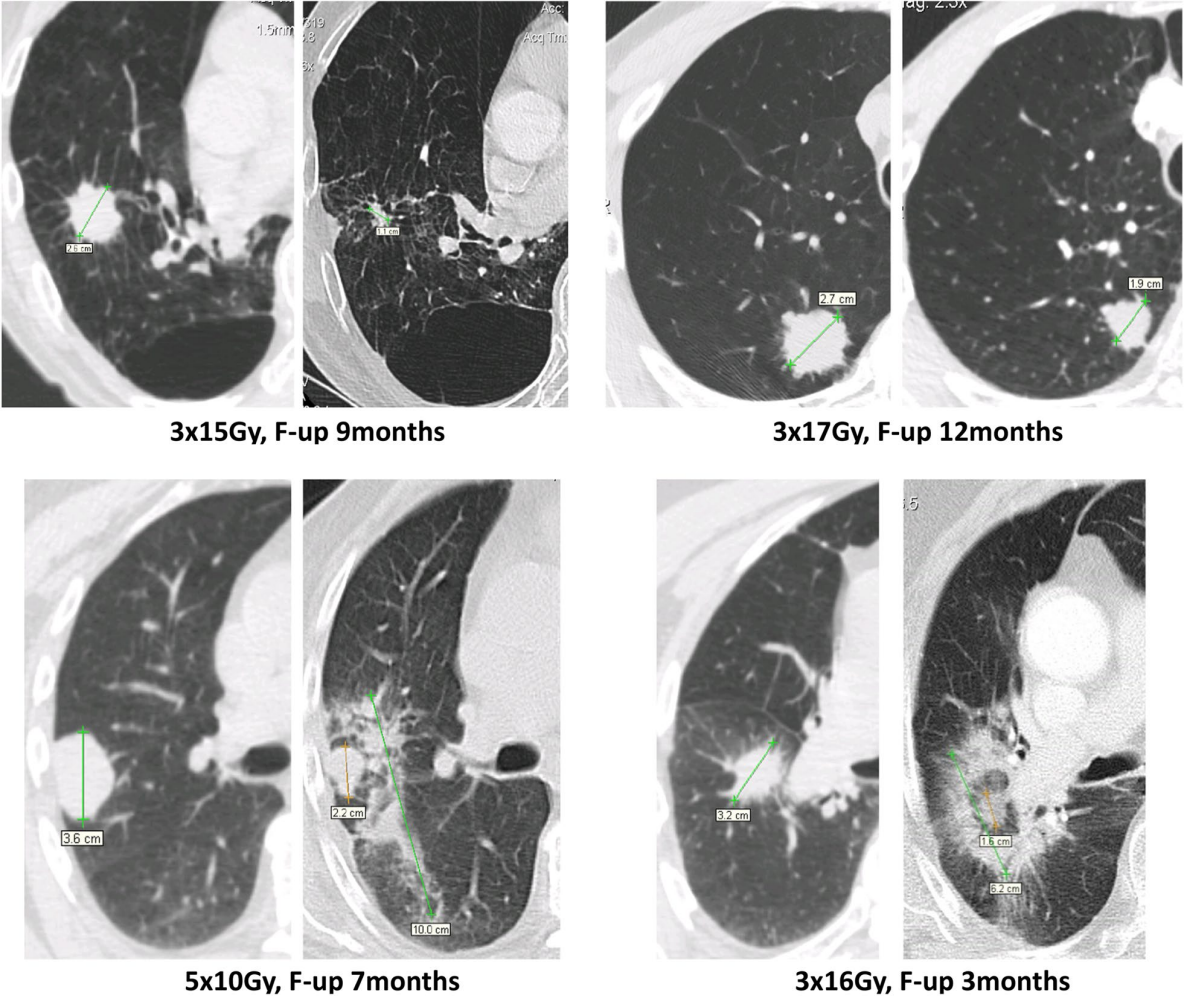
Representative chest CT images of patients who did not (upper row) or did (lower row) develop local lung injury induced by robotic stereotactic body radiation therapy of early-stage non-small cell lung cancer

Clinical and radiological follow-up including chest CT scans was scheduled at 3 and 6 months after radiotherapy and every 6 months thereafter. A local recurrence was assumed if the irradiated lesion showed a solid core that increased by at least 25% compared to the last follow-up and exhibited further growth. The first occurrence of diffuse or patchy consolidation, diffuse or patchy ground glass opacity or modified or mass like consolidation in the lung tissue adjacent to the tumor was regarded as radiation induced lung injury (termed local fibrosis, LF, see Fig. 1) and recorded as an event with regard to the time interval to the date of first irradiation [41, 42]. Lung tissue changes smaller than the original tumor, scar-like patterns distant to the tumor and lung toxicities without clear spatial or temporal relation to radiotherapy (early acute pneumonia, late acute pneumonia, pneumonitis spatially not correlated to the PTV) were not considered. In cases where a growing lesion could not be differentiated from local fibrosis, an FDG-PET-CT scan or a biopsy was performed in order to confirm or reject the diagnosis of a local recurrence.

### Image processing and feature extraction

Image processing was performed using Python 3.6.7 (Python Software Foundation, Beaverton, Oregon, USA). The original DICOM data containing manual delineations of the gross tumor volume (GTV) and anatomical image data were restored from the Cyberknife^R^ archive and subsequently used to extract the target volumes for radiomic analysis. For all further image processing, the software package pyradiomics 2.0.1 [43] was used that allows the extraction of standardized features which were defined by the IBSI (Image Biomarker Standardization Initiative) [44]. Preprocessing included resampling of the CT images and masks to isotropic voxels of 1 mm^3^ by the standard procedures of pyradiomics (B-spline interpolation for the CT images and nearest-neighbour interpolation for the binary masks) and removal of all voxels with Hounsfield units (HU) below (− 400) HU and above 1000 HU from the volume which were assumed to represent normal lung and bony tissue unintentionally included in the GTV by manual segmentation. Radiomic features were calculated based on the original image and after wavelet filtering, yielding eight additional image types based on the application of wavelet-based high-pass or low-pass filters to each of the three dimensions. In addition to 14 features descriptive of the target's shape, 93 features were calculated for each of the nine image types, resulting in a total of 851 radiomic features.

### Model development and statistical analysis

All model development was performed on the training cohort and the model parameters were optimized using cross-validation schemes. First, the primary set of radiomics features was reduced by identifying and removing linearly correlated features with a Pearson correlation coefficient > 0.90. Out of the 851 extracted features, 652 were found to be highly linearly correlated and removed from the analysis. The remaining 199 features were z-normalized and used to develop predictive models for each of the four endpoints: LC, OS, DFS and occurrence of local lung fibrosis (LF) after SBRT. Two different models implemented in the scikit-survival package for Python [45] were applied. The (linear) Cox Proportional Hazard model was used in conjunction with elastic net regularization of the feature coefficients (Coxnet, [46]) by means of a grid search for the optimal penalty parameter (alpha) with 10-times repeated fivefold random cross validation within the training set. Thus, the algorithm tries to reduce as many feature coefficients as possible to zero, and to achieve good predictions from the resulting smaller selection of features in the 10 × 5 validation data sets each comprising 20% of the training data. The model with the best cross-validated average training performance was then re-evaluated both in the complete training and the test set, where the feature values of the test set were subjected to the z-transformation parameterized from the training set. In order to allow for complex non-linear relationships between feature values and treatment outcome, a gradient-boosted ensemble of 100 regression trees (Gradient Boost, [47]) with the partial likelihood loss of the Cox’s proportional hazards model used as the loss function was also chosen as a model. In the training set, the main parameters of the model (learning rate, dropout rate and subsampling rate) were optimized by a 10-times repeated fivefold grid search with cross-validation. Thus, an intermediate model that returns a list of features and  their importance was generated. The list was then used to build models with increasing number of features, by starting with the one feature with the largest importance and successively adding the next important ones. At each step, the model was retrained and cross-validated (10-times repeated fivefold cross validation) in the training set. Typically, the cross-validated performance increased initially, but decreased by including more and more features due to overfitting. As before, the model with the optimal cross-validated performance was applied to the feature-normalized training and test sets.

In addition to the radiomics features, the following continuous clinical and dosimetric variables were analyzed in univariate Cox regression models with respect to their potential impact on any of the endpoints in order to select them as predictive features: GTV, PTV, GTV-D_max_, GTV-D_mean_, GTV-D_95%_, PTV-D_95%_, Lung-D_1ml_, Lung-D_10ml_, Lung-D_50ml_, Lung-D_100ml_, tumor diameter, age and Charlson Comorbidity Score. Categorical clinical and treatment related factors were investigated using the Kaplan–Meier method and survival estimates were compared using two-sided log rank tests. These included: gender, T-Stage (T1 vs. T2), histology (squamous cell/ adeno /other/ unknown) and fiducial tracking (no/ yes).

**Fig. 2:**
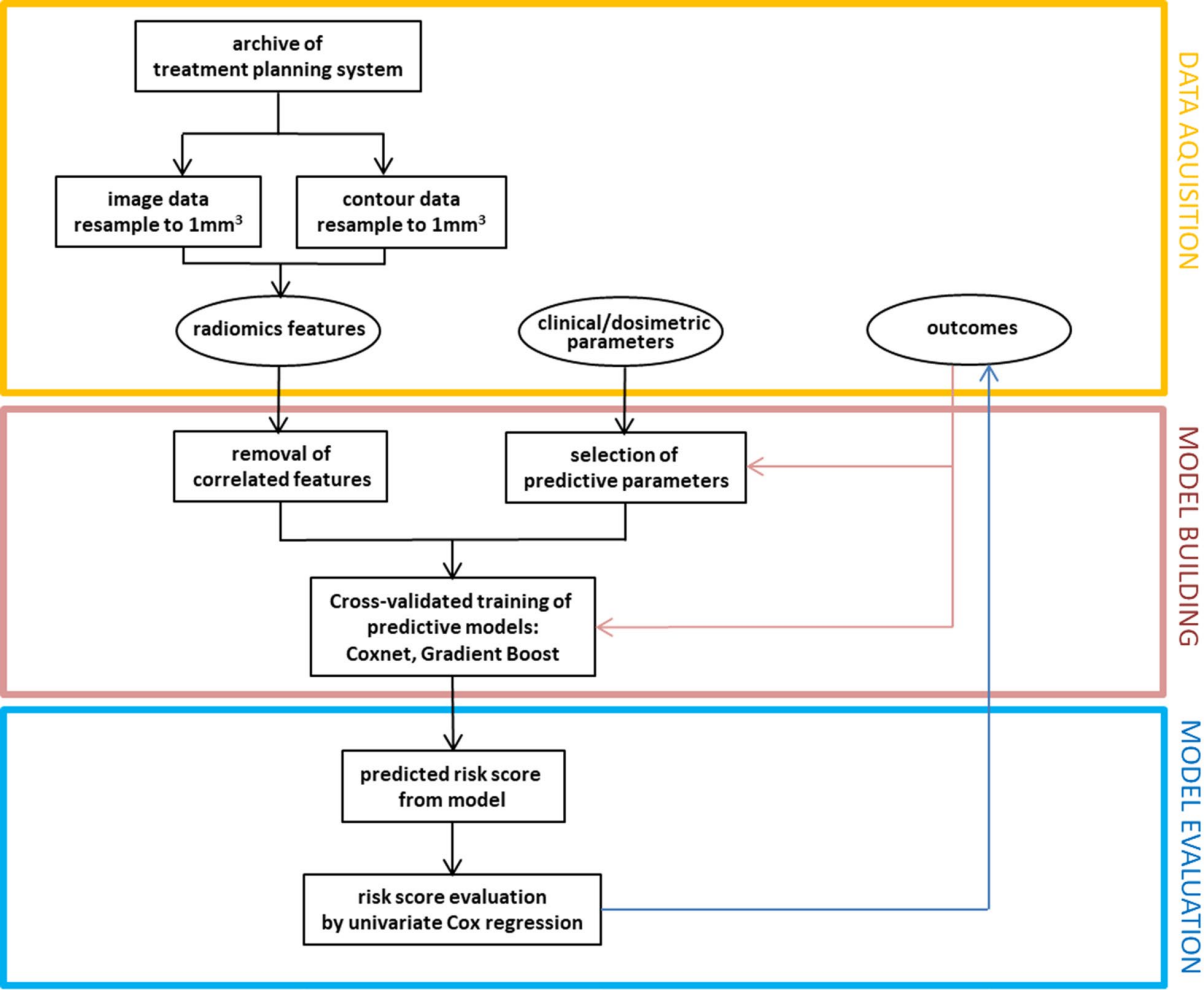
Workflow for generating and validating the developed models

For prediction of radiation-induced lung injury, Coxnet and Gradient Boost models were computed using only clinical/dosimetric features, only imaging features or both types of features. In case of the default settings for the Coxnet model used here, a baseline survival function is not calculated and the predictions are risk scores of arbitrary scale, while the scores of the Gradient Boost models built with the partial likelihood loss of the Cox’s proportional hazards model used as the loss function can be interpreted as log hazard ratios. The performance of any model was evaluated in the test set by means of the concordance index and the significance level of the predicted risk score when used as a continuous variable in a univariate Cox regression (p_Cox_). For purposes of illustration, the risk scores were dichotomized by their median and the Kaplan–Meier curves for the resulting low- and high risk groups were depicted. All statistical analyses were performed with the Lifelines python package (version 0.25.10, https://doi.org/10.5281/zenodo.4579431) and cross-checked by SPSS (vs. 24, Armonk, NY, USA). A *p*-value of < 0.05 was considered significant. The complete workflow is depicted in Fig. 2.

## Results

### Clinical outcome

**Fig. 3:**
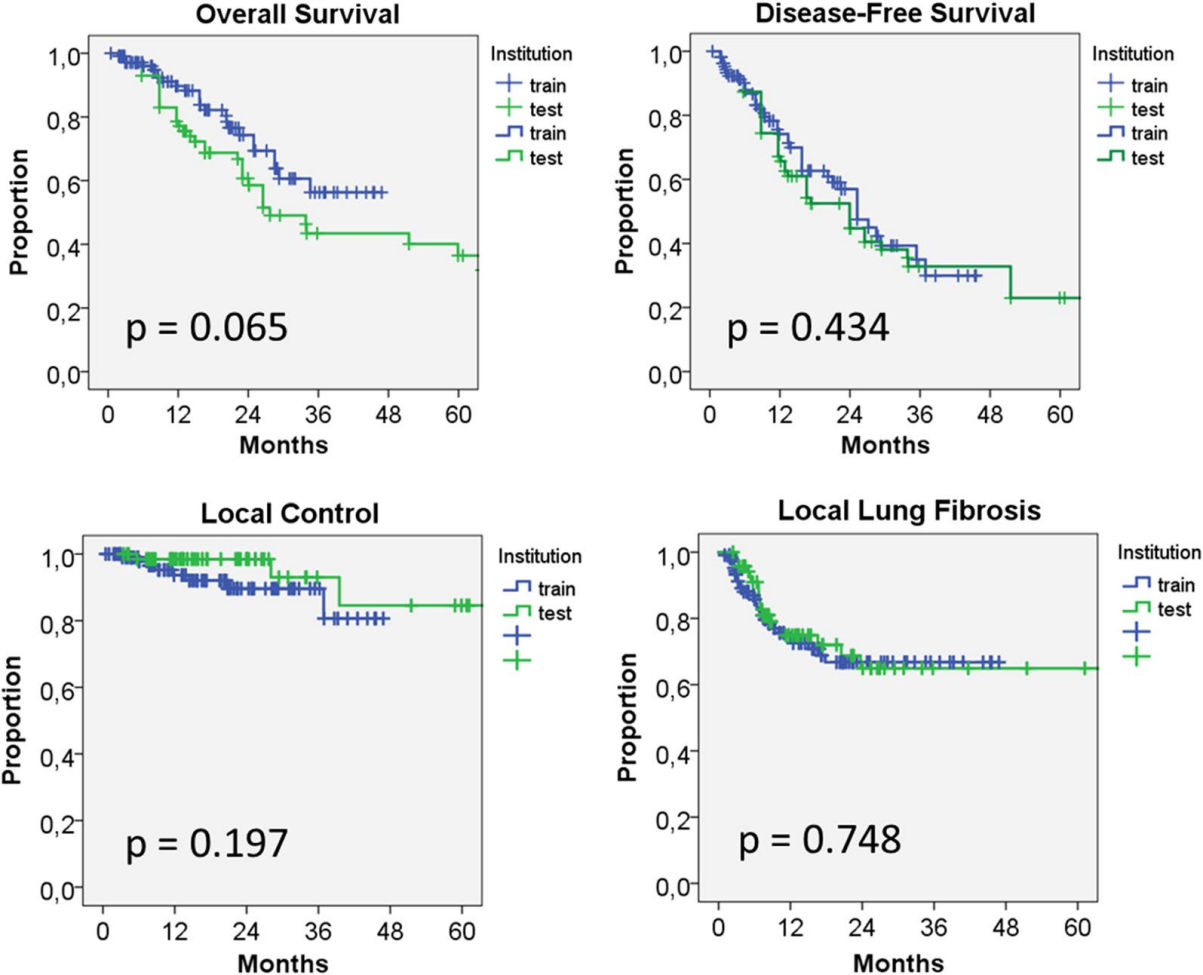
Kaplan-Meier curves for overall survival (OS), local control (LC), disease free survival (DFS) and occurrence of local lung fibrosis after SBRT for the training and testing cohort. No significant difference between the cohorts was measured for any endpoint

The outcome in terms of the analyzed clinical endpoints did not differ significantly between the two cohorts (Fig. 3). Overall survival at 36 months amounted to 56% versus 43%, *p* = 0.065), median DFS was 25 months versus 23 months, *p* = 0.43 and local control rates at 36 months were 90% vs. 93%, *p* = 0.197). In the training set, none of the clinical and dosimetric factors had a significant influence on these endpoints. Local lung fibrosis developed in 33% vs. 35% of the patients (*p* = 0.75), all events were observed within 36 months after irradiation. Three dosimetric factors (GTV_mean_, PTV-D_95%_, Lung-D_1ml_) and the patient's age had a significant impact (*p* < 0.05) on the development of local lung fibrosis with an increase in hazard of approximately 6% per Gy and per year of age.

### Radiomics models for OS, DFS and local tumor control

As shown in Table 3, the Coxnet model failed to substantially reduce the optimal feature number for these endpoints. Although highly predictive models (CCI 0.77–0.94, p_Cox_ < 0.005) were such found in the training set, these models showed poor cross-validation (CCI 0.52–0.54) and test set (CCI 0.36–0.49) performance. By application of the Gradient Boost model, a substantial feature set reduction to 5–22 features was achieved. However, despite reasonable cross-validation scores of 0.68–0.89, these models also failed to generalize to the test set. Due to the absence of any predictive models from clinical/radiological and radiomics features, combined models were not evaluated for these endpoints.

**Table 3:** Results of radiomics machine learning models for predicting overall survival, disease-free survival and local tumor control

Endpoint	Coxnet				Gradient boost			
	Number of features	CCI train-set	CCI cross-valid	CCI test-set	Number of features	CCI train-set	CCI cross-valid	CCI test-set
Overall survival	191	0.80 *p* < 0.005	0.52 ± 0.15	0.46 n.s	22	0.99 *p* < 0.005	0.68 ± 0.13	0.45 n.s
Disease free SV	197	0.94 *p* < 0.005	0.54 ± 0.11	0.49 n.s	10	0.97 *p* < 0.005	0.76 ± 0.09	0.52 n.s
Local control	199	0.77 *p* < 0.005	0.54 ± 0.24	0.36 n.s	5	0.98 *p* < 0.005	0.89 ± 0.11	0.17 n.s

*CCI* concordance index, means ± standard deviation are shown, *p* values: significance level of the model risk score in univariate Cox regression analysis.

### Clinical/dosimetric, radiomics and combined models for development of lung fibrosis

**Fig. 4:**
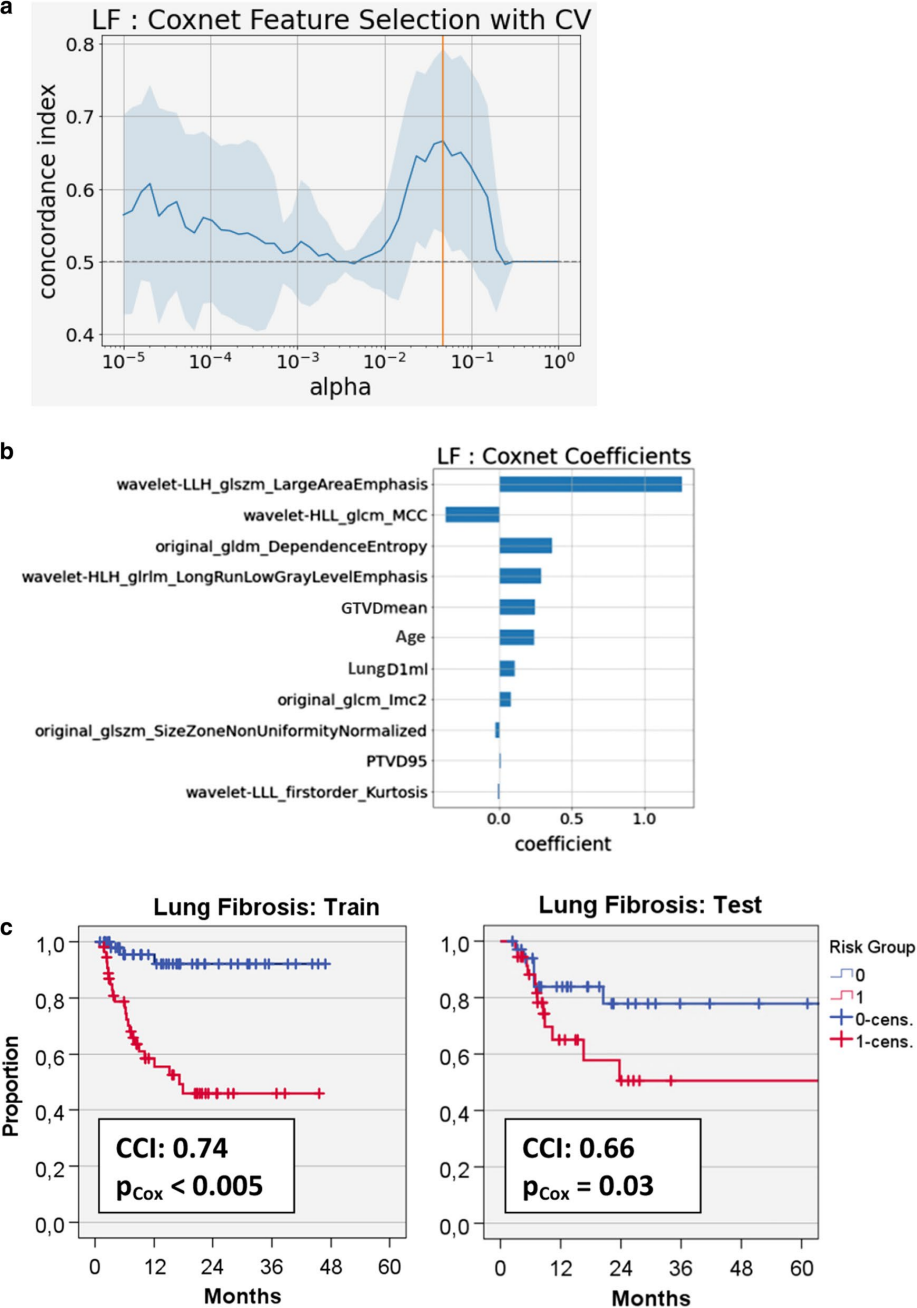
**a** Regularization and feature selection by repeated cross validation (CV) in a combined Coxnet model for development of lung fibrosis (LF) in the training set. The optimal model arose at an alpha-value of 0.5 × 10^–2^ where a mean concordance index (CCI) of 0.67 ± 0.12 was achieved. **b** Coefficients for the optimal Coxnet model that comprised 4 clinical/dosimetric and 7 radiomics features. **c** Kaplan–Meier curves displaying performance of the radiomics model in the training and test cohorts when stratifying patients into low and high risk groups by the respective medians of the model risk scores (train: 40.2, range 31.4–46.0; test: 42.4, range 25.0–60.4); pCox: Significance level for the model risk score used as a continuous variable in a univariate Cox regression analysis

Using the 4 identified clinical/dosimetric variables, both the Coxnet and Gradient Boost algorithms selected 3 of them (age, GTV_mean_ and Lung-D_1ml_) for building predictive models from the training set (CCI 0.71/0.73) which in case of the Coxnet also achieved a CCI of 0.65 (p_Cox_ = 0.04) in the test set (Table 4). Feature selection was also successful in both radiomics models where the Gradient Boost Model had a CCI of 0.59 (p_Cox_ = 0.02) using two features (Maximal Correlation Coefficient of the GLCM extracted from images filtered by two different wavelet kernels); this model could not be further improved by including any clinical/dosimetric variables. The best combined model resulted from the Coxnet that selected the 4 clinical/dosimetric variables and another 7 radiomics features and had a CCI of 0.66 (p_Cox_ = 0.03) in the test set (Fig. 4). The two radiomics features with the largest coefficients in this model were the Large Area Emphasis from the wavelet-filtered gray-level size zone matrix (GLSZM) and, as in the radiomics Gradient Boost model, the GLCM Maximal Correlation Coefficient from the wavelet-filtered images.

**Table 4:** Results of machine learning models for predicting local lung fibrosis

	Coxnet				Gradient boost			
Features	number of features	CCI train-set	CCI cross-valid	CCI test-set	Number of features	CCI train-set	CCI cross-valid	CCI test-set
Clinical/dosimetric	3^§^	0.71 *p* < 0.005	0.68 ± 0.11	0.65 *p* = 0.04*	3^§^	0.73 *p* < 0.005	0.64 ± 0.12	0.62 n.s
Radiomics	10	0.79 *p* < 0.005	0.64 ± 0.13	0.58 n.s	2^†^	0.75 *p* < 0.005	0.72 ± 0.11	0.59 *p* = 0.02*
Combined	4 + 7	0.74 *p* < 0.005	0.67 ± 0.12	0.66 *p* = 0.03*	0 + 2^†^	0.72 *p* < 0.005	0.72 ± 0.11	0.59 *p* = 0.02*

*CCI* concordance index, means ± standard deviation are shown, *p*-values: significance level of the model risk score in univariate Cox regression analysis

^§^Age/ GTV_MeanDose_/LungD_1ml_

^†^wavelet_HLH_glcm_MCC/wavelet_HLL_glcm_MCC (= GrayLevelCo-occurrence matrix maximal correlation coefficient)

## Discussion

### Summary of findings

In the present analysis, two cohorts of early-stage lung cancer patients treated with robotic stereotactic body radiotherapy at two different institutions were investigated. Although slightly different fractionation schedules were applied, oncologic outcome in terms of local tumor control, disease-free survival and overall survival were well comparable. Importantly, the frequency and time course of development of radiation-induced local lung injury was also similar in the two cohorts. Radiomics analysis based on a selected set of standardized features and state-of-the-art modelling in the training cohort resulted in models for prediction of radiation-induced local lung injury that performed well also in the test cohort. The predictive ability of the radiomics models resembled that of a model from a selection of clinical/dosimetric variables, but the marginally best performance was achieved in a model that combined a small number of clinical/dosimetric and radiomics features. However, the models for the endpoints of oncologic outcome (OS, DFS, local control) failed to generalize to the test cohort.

### Prediction of local radiation-induced lung injury

To the best of our knowledge, this is the first report that aimed at generating machine-learning models for the development of local lung injury from the GTV after lung SBRT [36]. Radiation-induced local lung injury that finally develops into local lung fibrosis is a typical event after lung SBRT, although it remains asymptomatic in most cases. It is probably triggered by the release of inflammatory cytokines such as TGF-ß from the tumor which subsequently initiate an immunological response [48, 49]. At first sight, it seems far from obvious how a texture pattern detectable by radiomics could predict for this event. However, an association between a pre-therapeutic radiomics feature (LoG standard deviation) with the TGF-ß signaling pathway has recently been observed, and in the same report, a radiomics score was correlated with the amount of tumor infiltration by T-lymphocytes [50]. The view that image features correlate with the presence of immune-competent cells in lung tumor tissue is also supported by the observation that lung tumors characterized by low CT intensity and high CT heterogeneity exhibited a high CD3 (T-lymphocyte) infiltration, suggestive of an activated immune state [51]. Interestingly, the most predictive features found in the present analysis were related to the heterogeneity of the lung tumor, as the GLSZM Large Area Emphasis is a measure of the distribution of large area size zones where a greater value is indicative of more larger size zones and more coarse textures, and the Maximal Correlation Coefficient of the GLCM is a measure of complexity of the texture resulting in values approaching unity for flat, homogenous regions.

In the present report, one of the radiomics models slightly improved the pure clinical/ dosimetric model (Coxnet) for the development of local lung fibrosis while the other radiomics model (Gradient Boost) did not benefit from the potential inclusion of the clinical/dosimetric features. In a comparable approach that has been applied for prediction of radiation pneumonitis from features of the total lung tissue in lung cancer patients treated with intensity-modulated radiotherapy (IMRT), the radiomics features also only slightly improved the predictive value of the model when added to clinical and dosimetric factors [47]. Interestingly, the inhomogeneous dose distribution usually generated by robotic radiosurgery and volumetric arc therapy has itself been analyzed with respect to dose distribution patterns (“dosiomics”) which in turn have been found to predict the incidence of radiation pneumonitis [52]. Thus, a more comprehensive model of radiation-induced lung injury could probably be built from incorporating texture analysis of the tumor, a shell [53, 54] comprising the adjacent lung tissue and the dose distribution.

### Prediction of local control, disease-free survival and overall survival

Although the two cohorts resembled each other in terms of oncologic outcome, the radiomics models did not generalize from the training to the test cohort with respect to these endpoints. In case of the Coxnet model that utilizes a simple, linear combination of features values, a substantial reduction of the feature space was not possible even during cross-validation within the training set itself. This is probably due to the fact that in the present cohort of inoperable patients with small lung tumors, local control could be achieved in most such that only a small number of events was available for training, and DFS and OS were largely independent from single radiomic properties of the tumors and confounded by other factors. Such, the predictive models for these endpoints were only found by chance and due to overfitting by using almost all features available. The Coxnet model used an ensemble of regression trees that can account for complicated, non-linear relationships between features and time to survival or other endpoints. Much higher cross-validation scores were found from a lower number of features, but the principle obstacles of relating confounded or low event-populated endpoints to a small set of radiomic features in an independent test set remained. As discussed above, the relation between selected radiomic tumor features, dose distribution and development of local lung fibrosis seemed be much stronger such that the type of the applied model became less important.

A compilation of recent studies on the impact of radiomics features on oncologic outcome for lung cancer patients after SBRT is presented in Table 5. Most of the studies applied single institution cross-validation or validation by test sets from the same institution and were able to predict local tumor recurrence, regional/nodal recurrence, distant failures and overall survival with a moderate accuracy. Of note, one report failed to observe features predictive of local recurrence [24]. Only in a minority of series were the results validated in test sets from independent institutions. In a large study from the Cleveland Clinic (Ohio, USA), a convolutional neural network (CNN) was trained to predict local recurrence in a group of > 900 lung cancer patients treated by SBRT. The stratification resulted in two groups with highly significant different risk for recurrence in both the training and test set [55]. Also in another study where a CNN was applied to both CT and PET images, a highly accurate classification of survival probability was achieved in an independent data set [21]. These results suggest that the application of CNN's that learn the relevant features for time-dependent oncologic predictions may be more effective than training models on predefined features [56, 57].

**Table 5:** Reports on outcome prediction of SBRT in lung cancer from analysis of radiomic features

Author	N	Modality/features (software applied)	^#^ features selected	Model type	Outcome measures	Validation	Result/comment
Huynh [24]	113	CT:1605 (in-house software)	12 + clinical	Survival analysis, cc-index	Recurrence, Distant mets., OS	Single institution cross validation	Risk for recurrence: no significant features Risk for dist. metastases: 1 sign. Feature OS: 4 significant features, cc = 0.67
Li [28, 29]	92	CT: 219 (Definiens Developer)	8–68 + clinical + semantic	ROC-analysis	Recurrence, RFS, OS	Single institution cross-validation	Risk stratification: AUC = 0.69–0.75
Zhang [58]	112	CT: 30 (ProCanVAS)	dependent on model	8 models: Random forest GLM, SVM etc	Recurrence, Distal failure, OS	Single institution cross validation	Risk stratification: AUC = 0.60–0.77
Yu [34]	442	CT: 12 (IBEX)	2	Random survival forests	Regional recurrence, OS	Single institution test set: 67%	OS risk stratification: *p* = 0.017 Recurrence risk stratification: *p* < 0.05 2 sign. features: kurtosis, homogeneity
Li [30]	110	CT + FDG-PET (learned by model)	from model	Kernelled support tensor machine	Distant failure	Single institution test set: 30%	Risk stratification: AUC = 0.80
Oikonomou [31]	150	CT + FDG-PET 2 × 21 (ProCanVAS)	6–8, 4 from PCA	PCA, logistic regression	Local control, Distant control, DSS, OS	Single institution cross validation	Risk stratification: *p* = 0.004–0.02 features: heterogeneity and morphology
Starkov [32]	116	CT: 2D-textures from solid core and GGO	2–30	Cox regression lasso	PFS, distant failure	Single institution cross validation	Risk stratification: *p* = 0.03 dependent on wavelet filtering
Lafata [26]	70	CT: 43	2	Logistic regression regularized	Local recurrence	none	Risk stratification: *p* = 0.048 features: density
Franceschini [23]	102	CT: 41 (LifeX)	4–6	Cox regression elastic net, back selection	Nodal relapse, PFS, DSS	Single institution Test set: 32%	Nodal Relapse: accuracy = 85% PFS: 53 vs.45 months features: heterogeneity
Lou [59]	944*	CT	learned by model	CNN, Multivariate competing risk	Local recurrence	**Multi institution test set: 10%**	Risk stratification: *p* < 0.002
Baek [21]	122	CT + FDG-PET 2 × 55,296	Features from k-medoids pool	CNN (U-Net) logistic regression	OS	**Independent institution test set: 21%**	Risk stratification: AUC = 0.87

*ProCanVAS* prostate cancer visualization and analysis system, *PCA* principal component analysis, cc-index: concordance index, *RFS* Recurrence-free survival, *ROC* receiver-operator-characteristics; validation by independent test sets shown in bold

*Includes recurrent lung cancers and pulmonary metastases

^#^Features from PET and CT

## Limitations of the present study

The present study, although based on the results of two independent cohorts, probably still lacks a sufficient number of patients and events needed for an informative analysis of the interaction between dosimetric parameters and radiologic tissue characteristics for prediction of local events (recurrence, local lung fibrosis) after SBRT of NSCLC. Also, the classification of local lung injury and tumor control is purely image-based and remains somewhat ambiguous, as tissue specimens are rarely available following SBRT. Differences in therapeutic strategies for detecting and treating metastases may have prevented the creation of a general radiomics-based model for prediction of DFS and OS.

## Conclusion

The present analysis provides evidence that radiomics analysis can, in principle, be used for prediction of local lung injury after SBRT of NSCLC in independent data sets and as such complements existing results on the successful prediction of other oncologic endpoints in this setting.

## Data Availability

The datasets generated and/or analyzed during the current study are not publicly available due but are available from the corresponding author on reasonable request.

## Abbreviations

NSCLC: non-small cell lung cancer
SBRT: stereotactic body radiation therapy
RILI: radiation-induced lung injury
UICC: Union for International Cancer Control
LC: local control
DFS: disease-free survival
OS: overall survival
LF: lung fibrosis
GTV: gross tumor volume
PTV: planning target volume
GTV-D_mean_: mean dose in GTV
GTV-D_max_: maximal dose in GTV
PTV-D_95%_: dose covering 95% of the PTV
GTV-D_95%_: dose covering 95% of the GTV
Lung-D_1ml_: dose achieved in 1 ml of the lung volume
Lung-D_10ml_: dose achieved in 10 ml of the lung volume
CT: computed tomography
PET: positron emission tomography
FDG-PET: fluor-deoxyglucose-positron emission tomograpy
DICOM: digital imaging and communications in medicine
HU: hounsfield unit
IBSI: image biomarker standardization initiative
CNN: convolutional neural network
LoG: Laplacian-of Gaussian
TGF-ß: transforming growth factor beta
CD3: cluster of differentiation (protein) 3
GLCM: gray-level co-occurrence matrix
GLSZM: gray-level size zone matrix
Coxnet: Cox proportional hazard model with elastic net regularization
Gradient Boost: gradient-boosted ensemble of regression trees
